# Associations between Choriocapillaris Flow on Optical Coherence Tomography Angiography and Cardiovascular Risk Profiles of Patients with Acute Myocardial Infarction

**DOI:** 10.3390/jpm12050839

**Published:** 2022-05-20

**Authors:** Dae Sung Kim, Byung Sik Kim, Heeyoon Cho, Jeong-Hun Shin, Yong Un Shin

**Affiliations:** 1 Department of Ophthalmology, Hanyang University Guri Hospital, Hanyang University College of Medicine, Guri-si 11923, Korea; 21crainstorm@naver.com (D.S.K.); hycho@hanyang.ac.kr (H.C.); 2 Division of Cardiology, Department of Internal Medicine, Hanyang University Guri Hospital, Hanyang University College of Medicine, Guri-si 11923, Korea; fish3777@hanmail.net

**Keywords:** acute myocardial infarction, cardiovascular risk, choriocapillaris, OCT-angiography

## Abstract

We evaluated the association between macular perfusion assessed via optical coherence tomography angiography (OCTA) and the cardiovascular risk profiles of patients with acute myocardial infarction (AMI). Patients with AMI who underwent comprehensive ophthalmological examinations and retinal OCTA were evaluated retrospectively. Retinal OCTA was performed for each patient within 3 days of admission. Quantitative analyses of the vessel density (VD) of the superficial capillary plexus (SCP) layers, deep capillary plexus layers (DCP), and choriocapillaris were performed after image processing. The 10-year risk of atherosclerotic cardiovascular disease (ASCVD), Global Registry of Acute Coronary Events (GRACE) score, reduction of atherothrombosis for continued health (REACH) score, and thrombolysis in myocardial infarction (TIMI) risk score were assessed. Sixty patients were included. VD in SCP and DCP was not associated with a 10-year ASCVD risk; however, choriocapillaris flow void features were significantly correlated with the 10-year ASCVD risk: decreased count, increased average size, and increased signal void area were correlated with increased 10-year ASCVD risk, GRACE score, REACH score, and TIMI risk score. In the multivariate analysis, a 10-year ASCVD risk (adjusted odds ratio [OR], 1.04; 95% confidence interval [CI], 1.01–1.08) and brain natriuretic peptide (adjusted OR, 1.00; 95% CI, 1.00–1.01) were significantly associated with the highest tertile of the average size of the choriocapillaris. Choriocapillaris flow void features measured using OCTA were associated with cardiovascular risk profiles in patients with AMI. OCTA may be used as an indicator of cardiovascular risk profiles and could improve cardiovascular risk assessments.

## 1. Introduction

Atherosclerotic cardiovascular disease (ASCVD) remains the leading cause of morbidity and mortality globally despite the substantial improvement in ASCVD outcomes in recent decades [1]. This is attributed to the suboptimal implementation of prevention strategies and uncontrolled ASCVD risk factors in many adults [2]; therefore, assessment of ASCVD risk remains the foundation of primary prevention [3]. Furthermore, since ASCVD recurrence and cardiovascular mortality risk are high in patients with experienced ASCVD, estimating the accurate cardiovascular risk and secondary prevention of cardiovascular events is also crucial [4].

The retina is a unique site where in vivo microvasculature can be directly visualized [5,6]. The underlying concept is that changes in the retinal vasculature may reflect cardiovascular disease risk; moreover, several studies have investigated the association between retinal microvasculature and cardiovascular risk profiles via optical coherence tomography angiography (OCTA)—a novel and non-invasive method for the visualization of retinal microperfusion states [7,8,9]. OCTA allows an accurate quantitative reflection of the in vivo state of retinal vessels and visualization of vascular density; however, little is known about the relationship between OCTA parameters and cardiovascular risk profiles. We, therefore, evaluated the vascular layer of the entire retina using OCTA to determine the association between macular perfusion and cardiovascular risk profiles of patients with acute myocardial infarction (AMI).

## 2. Materials and Methods

### 2.1. Study Design

This was a retrospective, single-centre study conducted at a tertiary referral centre. We reviewed the medical records of patients hospitalized for AMI. A total of 463 consecutive patients with AMI were hospitalized and underwent coronary angiography between 1 January 2018, and 31 December 2019, at our hospital. AMI included ST-elevation myocardial infarction (STEMI) and non-ST-elevation myocardial infarction (NSTEMI), defined according to practical guidelines [10,11]. Ophthalmic examinations were performed only for screening of retinal vascular diseases such as retinal vein occlusion or when hospitalized patients complained of any visual symptoms and consented to the examination.

**Figure 1:**
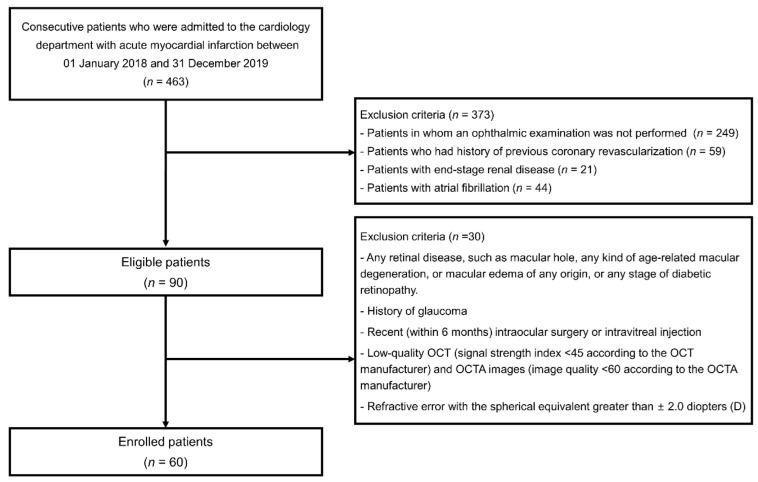
Flow chart of enrolled patients and exclusion.

The inclusion criteria were the availability of OCT and OCTA images with unremarkable media opacity. When both eyes met the inclusion criteria, the eye with the best-corrected visual acuity (BCVA) was selected. Retinal OCTA was performed for each patient within 3 days of admission. Patients who did not undergo ophthalmic examination because they did not want ophthalmic examination during the time of admission, patients who had a history of previous coronary revascularization, patients with end-stage renal disease, and patients with atrial fibrillation were excluded. The other exclusion criteria were as follows: any retinal disease, such as the macular hole, any kind of age-related macular degeneration, or macular edema of any origin, or any stage of diabetic retinopathy; a history of glaucoma; recent (within 6 months) intraocular surgery or intravitreal injection; low-quality OCT (signal strength index < 45 according to the OCT manufacturer) and OCTA images (image quality < 60 according to the OCTA manufacturer); refractive error with the spherical equivalent greater than ±2.0 diopters (D) (Figure 1).

The Institutional Review Board (IRB) of Hanyang University Guri Hospital approved this study design and was conducted in accordance with the tenets of the Declaration of Helsinki (IRB no. 2021-04-029). The IRB of Hanyang University Guri Hospital approved a waiver of informed consent due to the retrospective design.

### 2.2. Ophthalmic Examination

Each participant underwent ophthalmologic examinations, including BCVA, non-contact tonometry, and swept-source optical coherence tomography (SS-OCT) and OCT-angiography (DRI OCT Triton; Topcon Corporation, Tokyo, Japan). A technician performed OCT and OCTA examinations with the pupil in a dilated state. OCT was performed using a 12 mm × 9 mm scan protocol consisting of 256 B-scans, in which each consisted of 512 A-scans. Total retinal thickness, retinal nerve fiber layer thickness, ganglion cell–inner plexiform layer thickness, and choroidal thickness were obtained through the layers automatically segmented by the image viewer (IMAGEnet 6 Version 1.28.17646; Topcon Corporation, Tokyo, Japan). The image of each patient was verified with manual error correction.

### 2.3. OCTA Image Acquisition

OCTA scans a macular cube with a size of 4.5 mm × 4.5 mm (equivalent to 15° × 15°), each cube contains 320 B-scans repeated four times. The scan size may vary depending on the axial length of each patient; however, the OCT device (Topcon Corporation, Tokyo, Japan) was set to 24.39 mm by the manufacturer. The division of the retina by layer was confirmed through auto segmentation of the image viewer; if it was divided differently from the standard, it was adjusted manually. The definitions of each en-face slab are as follows; the superficial capillary plexus (SCP): from 2.6 μm below the internal limiting membrane to 15.6 μm below the interface of the inner plexiform layer and inner nuclear layer (IPL/INL); the deep capillary plexus (DCP): from 15.6 μm below the IPL/INL to 70.2 μm below the IPL/INL; and the choriocapillaris layer (10–40 μm below the retinal pigment epithelium-fit segmentation) [12].

### 2.4. OCTA Image Analysis

**Figure 2:**
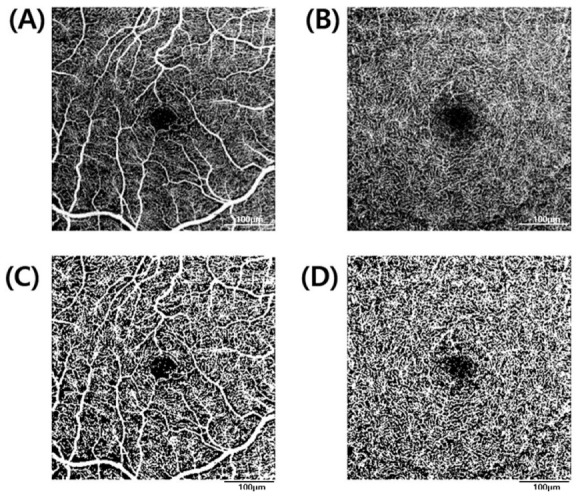
Measurement of the vascular density of the superficial and deep retinal layer. (**A**) Superficial retinal layer angiogram. (**B**) Deep retinal layer angiogram. (**C**) Binarization of the superficial retinal layer using “Phansalkar local thresholding.” (**D**) Binarization of the deep retinal layer using “Phansalkar local thresholding”.

**Figure 3:**
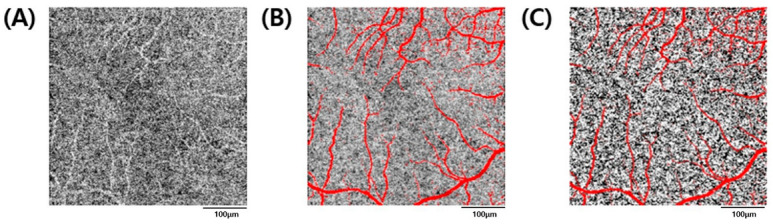
Measurement of the choriocapillaris flow void features. (**A**) Choriocapillaris angiogram. (**B**) Projection mask (red pixels) derived from the superficial and deep angiograms superimposed on the choriocapillaris angiogram. (**C**) Binarization of choriocapillaris layer using the “Otsu method”.

Fiji (version 1.53f51; National Institutes of Health, Bethesda, MD, USA), an open-source image processing software, was used to obtain the perfusion-related parameters for each retinal layer. After exporting en-face images through the OCT viewer, the superficial and deep retinal layers were binarized through “Phansalkar local thresholding.” In the binarized image, the blood vessels were shown as white objects, after inverting the image into black and white, particle analysis was performed to calculate the vessel density (VD) (Figure 2). In the choriocapillaris, the “Otsu method” was used for binarization, and perfused vessels appear as white objects and the background as black. The large blood vessels of the superficial retinal layers (Threshold 220 or higher) were masked, followed by particle analysis. Flow voids are areas where the flow signal becomes lower when the flow of choriocapillaris is measured by OCT angiography. In other words, the flow void refers to the non-white part of the binarized image, which can be considered as the non-flow part of the choriocapillaris. Particle analysis using ImageJ provides the following three basic parameters: the number of particles, the area occupied, and the average size of the particles. After flipping the image while binarizing to see the flow void, an analysis was performed. Flow void count, the signal void of the deficit area, and the average size of the flow void area were measured by this method (Figure 3) [13].

Two ophthalmologists (D.S.K and Y.U.S) reviewed the OCT and OCTA images, checked and corrected segmentation errors before image processing, and excluded images from analysis if there were severe artifacts after image processing. The vascular density of the SCP and the DCP was automatically measured during image processing using Image J, but CCP measurements required data processing to adjust thresholds for masking large vessels in the SCP layer to remove projection artifacts; therefore, each investigator processed one image twice using Image J and averaged the values. In addition, the measured values of the two researchers were averaged and analysed. We calculated the intraclass correlation coefficients to assess the agreement between the CCP measurements.

### 2.5. Clinical and Laboratory Evaluation

The patients’ demographic and clinical characteristics, including age; sex; blood pressure; heart rate; Killip class; smoking status; and comorbidities such as hypertension, diabetes mellitus, dyslipidemia, chronic kidney disease, and ischemic stroke were obtained from the participant’s medical records. Laboratory test results for troponin I, brain natriuretic peptide (BNP), blood glucose levels, lipid profiles, creatinine, and estimated glomerular filtration rate (eGFR) were recorded; moreover, the carotid arteries were evaluated by an experienced medical sonographer using commercially available ultrasound equipment (IE33; Philips Healthcare, Andover, MA, USA) with an 11-MHz linear array probe. Intima-media thickness (IMT) in the common carotid arteries was assessed in the far wall according to the leading-edge principle using semi-automated edge-detection software. The mean carotid IMT in both the carotid arteries was measured. The presence of carotid plaque was defined as a focal thickening of the IMT > 1.5 mm or >50% of the surrounding wall [14]. Transthoracic echocardiography was performed using the same ultrasound system (IE33; Philips Healthcare, MA, USA) with an S5-1 transducer. Left ventricular ejection fraction (LVEF) was assessed using the modified biplane Simpson’s method [15]. Brachial-ankle pulse wave velocity (baPWV) was measured using an oscillometric sphygmomanometric device for non-invasive evaluation of arterial stiffness (VP-1000 plus; Omron Colin, Kyoto, Japan) [16]. The mean values of the left and right baPWV were used.

### 2.6. Cardiovascular Risk Scores

The cardiovascular risk scores of the participants were calculated. The 10-year risk for preventable ASCVD events (including coronary death, nonfatal myocardial infarction, and fatal or nonfatal stroke), a critical component of the ASCVD risk reduction approach in clinical practice guidelines for primary prevention [3], was calculated. The Global Registry of Acute Coronary Events (GRACE) score, which is recommended for risk stratification in AMI and known to predict in-hospital mortality, was calculated [11]. In addition, we calculated the Thrombolysis in Myocardial Infarction (TIMI) risk score, which is associated with short-term mortality of patients with AMI [17] and the residual Synergy Between Percutaneous Coronary Intervention with Taxus and Cardiac Surgery (SYNTAX) score, a grading system that evaluates the complexity and prognosis of patients undergoing percutaneous coronary intervention [18]. Finally, we calculated the risk score for secondary prevention with the Reduction of Atherothrombosis for Continued Health (REACH) score, which is associated with the risk of recurrent cardiovascular events (cardiovascular death and subsequent events within 2 years of follow-up) [19].

### 2.7. Statistical Analyses

Data are presented as mean ± standard deviation for continuous variables and as frequency (percentage) for categorical variables. First, analysis was performed to determine the most appropriate OCTA parameters that might reflect cardiovascular risk. The study participants were divided into tertiles according to the 10-year ASCVD risk, and the differences in OCTA parameters and clinical variables between the groups were compared. Continuous variables were compared using a one-way analysis of variance with Tukey’s post hoc test for normally distributed variables and Kruskal–Wallis test, followed by Dunn’s multiple comparisons for non-normally distributed variables. Categorical variables were compared using the chi-square test or Fisher’s exact test; moreover, Pearson’s correlation was used to analyse the interrelationship between OTCA parameters and cardiovascular risk scores, including 10-year ASCVD risk, GRACE score, REACH score, and TIMI risk score.

Next, further analysis using the OCTA parameters, which showed the strongest correlation with the cardiovascular risk scores, was performed. The study participants were divided into tertiles according to the selected OCTA parameter, and the differences in clinical characteristics between the groups were compared. Multivariate logistic regression was used to evaluate independent factors associated with the selected OCTA parameter. Significant factors (*p* < 0.05) in the univariate model, except for variables reflected in the risk scores, were included in the multivariate logistic regression model. Backward elimination with a threshold of *p* = 0.1 was used as the criterion for retention of a variable in the model. Statistical significance was set at *p* < 0.05, and all analyses were performed using SPSS software (Version 26; IBM Corp, New York, NY, USA).

## 3. Results

### 3.1. Baseline Characteristics According to the 10-Year ASCVD Risk and Association between OCTA Parameters and Cardiovascular Risk Scores

**Figure 4:**
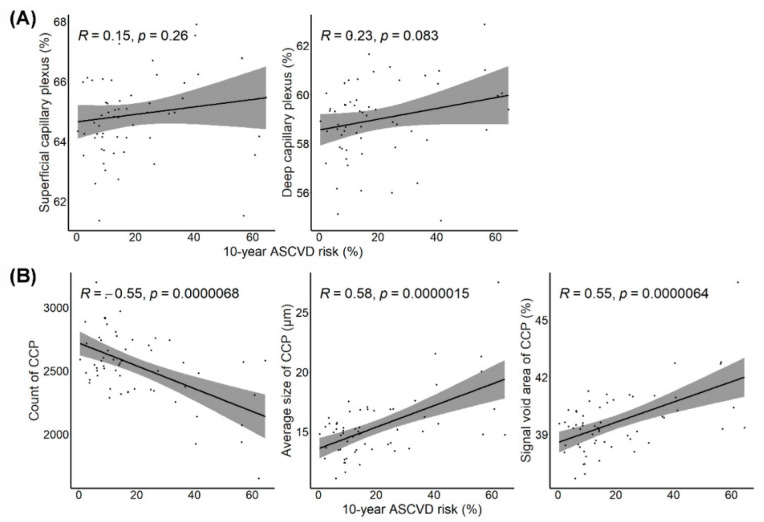
The association between the 10-year atherosclerotic cardiovascular risk and optical coherence tomography angiography parameters, including (**A**) retinal vessel density of the superficial capillary plexus and deep capillary plexus, and (**B**) choriocapillaris flow void features. CCP, choriocapillaris; ASCVD, atherosclerotic cardiovascular disease.

**Figure 5:**
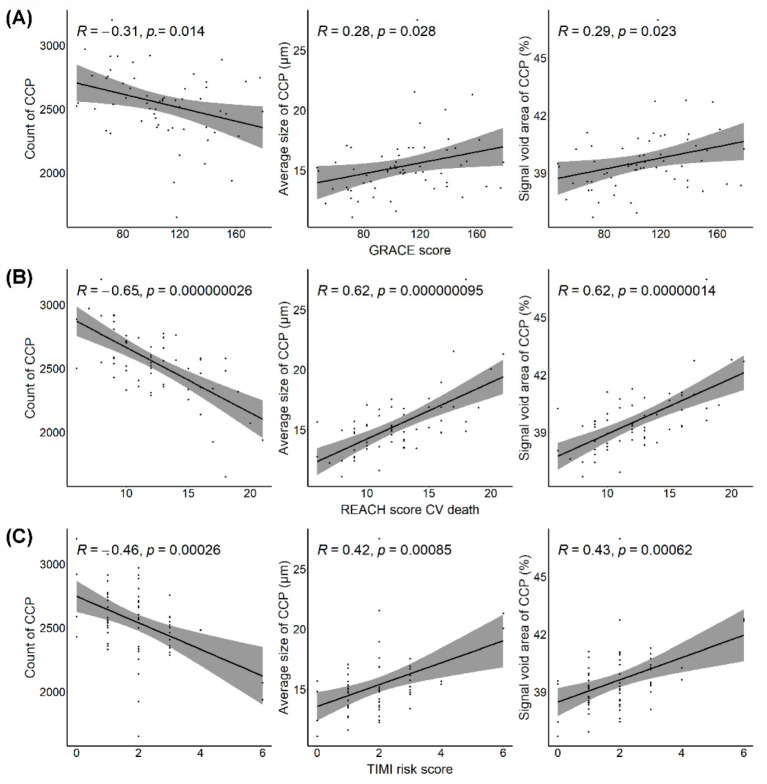
The association between choriocapillaris flow void feature measurement by optical coherence tomography angiography and cardiovascular risk scores. (**A**) GRACE score, (**B**) REACH score, (**C**) TIMI risk score. CCP, choriocapillaris; GRACE score, Global Registry of Acute Cardiac Events score; REACH score, Reduction of Atherothrombosis for Continued Health score; CV, cardiovascular.

A total of 463 patients with AMI were screened. Patients who did not want to undergo ophthalmic examinations (*n* = 249), those who had a history of previous coronary revascularization (*n* = 59), those with end-stage renal disease (*n* = 21), and those with atrial fibrillation (*n* = 44) were excluded. Of 90 eligible patients, only 60 were enrolled in this study after the application of ophthalmologic exclusion criteria (Figure 1). The mean age of the 60 patients was 59 ± 12 years, and 54 (90%) were men. Two investigators reviewed all OCT and OCTA images and did not find any artifacts in these images; therefore, the original OCT segmentation was not modified. For the CCP parameters, the repeatability of each investigator was high for all three CCP flow void features (intraclass correlation coefficient (ICC), 0.953–0.996 for Y.U.S and 0.966–0.998 for D.S.K). The reproducibility between two readers was also high (ICC, 0.911–0.957) in all parameters. Patients with a high 10-year ASCVD risk (third tertile) were significantly older; they had higher systolic blood pressure, history of diabetes and chronic kidney disease, and baPWV and more carotid plaque, and lower eGFR than patients with low 10-year ASCVD risk (first tertile); moreover, patients with high 10-year ASCVD risk had a significantly lower count of choriocapillaris flow voids, higher average size of flow void area, and signal void area of choriocapillaris than patients with low 10-year ASCVD risk; however, retinal VD in the SCP and DCP did not differ between the groups according to the 10-year ASCVD risk (Table 1). Figure 4 shows the association between OCTA parameters, including retinal VD in the SCP and DCP, and choriocapillaris flow void features and the 10-year ASCVD risk according to Pearson’s correlation. VD in SCP and DCP was not associated with a 10-year ASCVD risk; however, the choriocapillaris flow void features (count, average size, and signal void area) were significantly correlated with the 10-year ASCVD risk. As the 10-year ASCVD risk increased, the number of choriocapillaris flow voids decreased, and the average size of the flow void area and signal void area of the choriocapillaris increased. Choriocapillaris flow void features were also significantly correlated with other cardiovascular risk scores, such as the GRACE, TIMI, and REACH scores (Figure 5). Similar to the 10-year ASCVD risk, as the cardiovascular risk scores increased, the number of choriocapillaris flow voids decreased, and the average size of the flow void area and signal void area of the choriocapillaris increased.

### 3.2. Baseline Characteristics According to Average Size of the Choriocapillaris Flow Void Area

**Figure 6:**
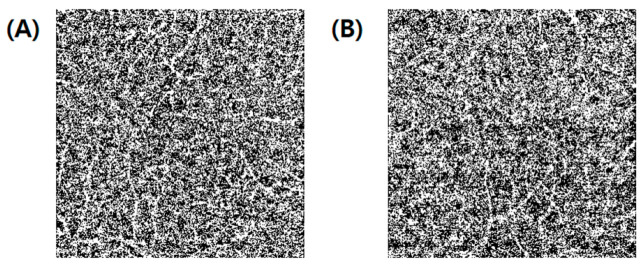
Representative optical coherence tomography angiography images of the choriocapillaris layer and choriocapillaris flow features in two patients with acute myocardial infraction with low and high cardiovascular risk scores, respectively. (**A**) Case #1 ASCVD 6.2, GRACE 76, count of CCP 2522, average size of CCP 15.22, signal void area of CCP 39.49. (**B**) Case #2 ASCVD 41.4, GRACE 179 count of CCP 2482, average size of CCP 15.67, signal void area of CCP 40.25. CCP, choriocapillaris; ASCVD, atherosclerotic cardiovascular disease; GRACE score, Global Registry of Acute Cardiac Events score.

Among the flow void features of the choriocapillaris, the average size of the choriocapillaris flow void area, which had the strongest correlation with the 10-year ASCVD risk (Pearson r = 0.580, *p* < 0.001), was selected for further analysis. We performed further analysis using this parameter by dividing it into tertiles. Table 2 displays the baseline characteristics according to the average size of the choriocapillaris flow void area. Patients with a high average size of the choriocapillaris flow void area (third tertile) were more likely to present with a high cardiovascular risk factor burden than patients with a low average size (first tertile); they were significantly older, had a history of hypertension and dyslipidemia, and had a high BNP level. Patients with a high average size of the choriocapillaris flow deficit area presented a more severe cardiovascular status with a 10-year ASCVD risk, GRACE, and TIMI risk scores, and REACH scores were significantly higher than the low average size of the choriocapillaris flow void area groups (Figure 6).

### 3.3. Associated Factors for the High Average Size of the Choriocapillaris Flow Void Area

Multivariate analysis using each cardiovascular risk profile and baseline characteristics with significance in the univariate model was conducted to evaluate the independent factors for the high average size of the choriocapillaris flow void area, except for variables reflected in risk scores. In the analysis with 10-year ASCVD risk, 10-year ASCVD risk (adjusted odds ratio [OR], 1.04; 95% confidence interval [CI], 1.01–1.08; *p* = 0.024) and BNP (adjusted OR, 1.00; 95% CI, 1.00–1.01; *p* = 0.044) were significantly associated with the highest tertile of the average size of the choriocapillaris flow void area (Table 3). Other cardiovascular risk profiles were also independently associated with a high average size of the choriocapillaris flow void area. In the analysis with GRACE score, GRACE score (adjusted OR, 1.02; 95% confidence interval [CI], 1.00–1.04; *p* = 0.042) and hypertension (adjusted OR, 6.76; 95% CI, 1.74–26.27; *p* = 0.006) were significantly associated with a high average size of the choriocapillaris flow void area (Table 4). In the analysis with REACH score, REACH score (adjusted OR, 1.41; 95% CI, 1.12–1.78; *p* = 0.003) was significantly associated with a high average size of the choriocapillaris flow void area (Table 5).

## 4. Discussion

Our study assessed the potential association between retinal microvasculature measured by optical coherence tomography angiography (OCTA) and cardiovascular risk profiles in patients with acute myocardial infarction (AMI). The major findings of this study were as follows: (1) Among OCTA parameters, choriocapillaris flow void features were significantly correlated with various cardiovascular scores that predict primary and secondary cardiovascular event risk, whereas vessel density (VD) in superficial capillary plexus (SCP) and deep capillary plexus layers (DCP) was not associated with cardiovascular risk scores. (2) As the cardiovascular risk scores increased, consistent changes in the choriocapillaris flow void features, such as decreased count of flow voids, increased average size of the flow void area, and increased signal void area were observed. (3) Within the average size of the choriocapillaris flow void area, which showed the strongest correlations with 10-year ASCVD risk among the flow void features of the choriocapillaris, patients with a high average size of the choriocapillaris flow void area were older, had more cardiovascular risk factors, and had higher risk scores than patients with a lower average size of the choriocapillaris flow void area. (4) The 10-year risk of ASCVD, GRACE, and REACH scores were independently associated with a high average size of the choriocapillaris flow void area, respectively.

Abnormalities of the retinal microcirculation, such as microaneurysms, arteriolar-venular nicking, and arteriolar narrowing independently predict future cardiovascular events [20]. Thus retinal vasculature may act as a predictive marker future cardiovascular risk; however, previous studies have largely used nonquantitative measures, such as clinical ophthalmoscopy, which is neither objective nor quantitative [21]. In the last few decades, digitized retinal photography, fundus fluorescein angiography, and other methods have been accepted as standardized and objective techniques for characterizing retinal microvasculature [22]. OCTA, which provides depth-resolved detailed images of retinal blood flow and choroid far exceeding those obtained with older forms of imaging and allows the quantitative evaluation of vascular parameters, has become recently available.

Data on the associations between systemic cardiovascular risk and OCTA parameters are limited. Particularly, previous data regarding the relationship between OCTA parameters and coronary artery disease were inconsistent. Arnould et al. reported that SCP density measured on OCTA was associated with the cardiovascular risk profiles [7]. In addition, Zhong et al. demonstrated that SCP and DCP were associated with the presence of coronary artery disease, while DCP was associated with the severity of coronary artery stenosis in patients with coronary artery disease [8]; however, SCP was not associated with hemodynamic variables in patients with myocardial infarction [9]. In our study, VD in SCP was not associated with cardiovascular risk profiles. The difference from the study of Arnould et al. could be attributed to the difference between the OCTA device (spectral domain-OCTA vs. swept source-OCTA) and the scanning protocol differences (3 mm × 3 mm vs. 4.5 mm × 4.5 mm) and the nature of the retinal blood flow. The retina has the intrinsic ability to maintain blood flow in various situations, called autoregulation [23]. In this study, patients without retinopathy were examined, and autoregulation of the retina worked normally, and no difference in was observed in VD between SCP and DCP. Racial differences may have contributed to the differences in the results of the two studies (Western in the study of Arnould et al. vs. Asian in our study). Another possible reason is that the proportion of patients with diabetes differed (22.8% in Arnould et al. vs. 3.33% in the present study). Even patients with diabetes who do not have retinopathy show vascular changes on OCTA [24]; therefore, we believe that the present study is the result of excluding vascular changes caused by diabetes as much as possible.

Wang et al. reported that decreases in choroidal VD were related to coronary artery and branch stenosis [22]. In acute hypertensive chorioretinopathy, Michiyuki et al. measured the choroid thickness using OCT and the choroidal blood flow rate through laser speckle flowgraphy, with a simultaneous increase in the choroidal blood flow velocity and thickness [25]. Kei et al. reported a negative correlation between KWB grade and choroidal VD in patients with hypertensive retinopathy [26]. Our study showed that a disturbed choriocapillaris flow on OCTA correlated with cardiovascular risk profiles. Choroidal status, such as choroidal thickness and choriocapillaris flow features, can be a biomarker of systemic diseases. Hemodialysis can cause changes in the choroidal thickness and choriocapillaris flow [27]. Spaide reported that age and hypertension could affect the choriocapillaris features on OCTA [28]. Conversely, Chua et al. suggested that high blood pressure might increase the blood flow of the choriocapillaris [29]. The exact mechanism by which systemic status can affect the choroid remains unclear. A possible explanation is that choroidal circulation is controlled primarily by the extrinsic autonomic system [30]. Since autonomic dysfunction is closely associated with atherosclerosis and cardiovascular disease, choroidal circulation may reflect cardiovascular risk [31]. Further studies are warranted to confirm the relationship between systemic and choroidal circulation.

The strength of this study is that it uses swept-source OCT to better see the choroidal status; moreover, because our study included patients hospitalized for AMI, despite its retrospective nature, comprehensive data on cardiovascular risk factors were obtained for all patients. To our knowledge, this is the first study to examine the choroidal flow patterns in patients with AMI. This study has some limitations. The first limitation is the retrospective nature of this study. Compared with a prospective study, the accuracy and completeness of the obtained data were insufficient. Second, we included patients with AMI who were hemodynamically stable and had undergone ophthalmological examination; therefore, unstable patients and those who did not undergo ophthalmologic examinations were excluded, which could have led to a selection bias. In addition, the study was performed in the acute phase of AMI. The systemic condition or treatment of the disease may therefore affect the OCTA parameters, and moreover, the 10-year ASCVD risk score may not be appropriate in these patients as it is used for purpose of primary prevention; however, we analyzed the association between OCTA parameters and various types of cardiovascular risk scores, such as the REACH score for secondary prevention or the GRACE score for risk stratification in patients with AMI. Third, calibration of the axial length is needed to accurately quantify OCTA parameters, but no axial length measurement data were available for this cohort; however, the low refractive errors of enrolled patients (spherical equivalents within ±2.0D) might minimize the axial length effect. Fourth, there may be a selection bias caused by excluding the cases with retinal diseases, such as diabetic retinopathy or age-related macular degeneration. We excluded these cases in this study because they were confusing factors for OCTA interpretation. Specifically, diabetic retinopathy can show retinal capillary changes on OCTA even in the early stages of DR [24]. In addition, in the cases of AMD, severe retinal structural deformation can cause OCTA artifact (e.g., segmentation errors), leading to inaccurate CCP measurement. In addition, a previous study reported that macular pathology, such as soft drusen, could cause choriocapillaris signal reduction and segmentation errors [32]. A future prospective study with larger samples and a further developed OCTA analysis technique is necessary to confirm the association between the CCP flow void features and acute MI. Finally, this single-center study comprised of a relatively small number of patients, which may not be representative of all patients with AMI and may raise issues related to statistical power in the analysis results.

## 5. Conclusions

In conclusion, we found that choriocapillaris measured using OCTA was associated with various cardiovascular scores that predict primary and secondary cardiovascular events. This study suggests the possibility and usefulness of OCTA as a novel tool for evaluating cardiovascular risk profiles, including primary and secondary prevention.

## Figures and Tables

**Table 1:** Baseline characteristics, including OCTA parameters in the 3 groups according to the 10-year ASCVD risk.

	Overall Patients (*n* = 60)	Low ASCVD Risk (*n* = 20)	Intermediate ASCVDRisk (*n* = 20)	High ASCVD Risk (*n* = 20)	*p*-Value
OCTA parameter					
Superficial capillary plexus	64.91 ± 1.41	64.66 ± 1.54	64.76 ± 1.07	65.31 ± 1.55	0.289
Deep capillary plexus	59.01 ± 1.63	58.48 ± 1.30	59.31 ± 1.54	59.23 ± 1.95	0.212
Choriocapillaris parameter					
Count	2544 ± 277	^†^ 2661 ± 212	^‡^ 2607 ± 225	2363 ± 299	0.001
Average size	15.34 ± 2.63	^†^ 14.29 ± 1.43	^‡^ 14.69 ± 1.69	17.06 ± 3.44	0.001
Signal void	39.60 ± 1.64	^†^ 38.96 ± 1.04	^‡^ 39.19 ± 1.18	40.63 ± 2.03	0.001
Clinical characteristics					
Age, years	59 ± 12	^†,^* 49 ± 8	^‡^ 58 ± 9	69 ± 11	<0.001
Male sex, *n* (%)	54 (90)	16 (80)	19 (95)	19 (95)	0.344
Systolic blood pressure, mmHg	136 ± 31	^†^ 121 ± 25	^‡^ 130 ± 29	157 ± 28	<0.001
Diastolic blood pressure, mmHg	83 ± 19	80 ± 16	80 ± 20	91 ± 19	0.118
Heart rate, beats/min	78 ± 23	76 ± 23	75 ± 19	83 ± 28	0.519
Current smoker, *n* (%)	37 (61.7)	11 (55)	16 (80)	10 (50)	0.112
STEMI, *n* (%)	37 (61.7)	14 (70)	14 (70)	9 (45)	0.172
Killip class	1.5 ± 1.0	1.7 ± 1.2	1.7 ± 1.2	1.2 ± 0.5	0.152
Comorbidities, *n* (%)					
Hypertension, *n* (%)	27 (45)	7 (35)	7 (35)	13 (65)	0.089
Diabetes, *n* (%)	17 (28.3)	3 (15)	4 (20)	10 (50)	0.029
Dyslipidemia, *n* (%)	14 (23.3)	4 (20)	3 (15)	7 (35)	0.404
Chronic kidney disease, *n* (%)	13 (21.7)	1 (5)	2 (10)	10 (50)	0.001
Ischemic stroke, *n* (%)	3 (5)	1 (5)	1 (5)	1 (5)	1
Laboratory findings					
Troponin I, ng/mL	3.2 ± 9.7	5.1 ± 14.8	3.1 ± 7.8	1.3 ± 2.2	0.477
BNP, pg/mL	260 ± 464	194 ± 201	126 ± 192	459 ± 726	0.053
Creatinine, mg/dL	0.98 ± 0.42	0.92 ± 0.40	0.95 ± 0.21	1.06 ± 0.58	0.552
eGFR, mL/min/1.73 m^2^	87 ± 20	^†^ 95 ± 23	87 ± 15	78 ± 20	0.038
Glucose level, mg/dL	168 ± 78	163 ± 59	178 ± 105	164 ± 63	0.798
Total cholesterol level, mg/dL	173 ± 43	184 ± 51	165 ± 28	171 ± 46	0.395
Triglyceride level, mg/dL	155 ± 68	166 ± 78	140 ± 60	158 ± 66	0.493
HDL-C level, mg/dL	41 ± 9	43 ± 8	39 ± 8	40 ± 10	0.37
LDL-C level, mg/dL	97 ± 34	106 ± 36	100 ± 25	87 ± 37	0.195
LVEF, %	51 ± 12	49 ± 9	52 ± 11	50 ± 16	0.719
baPWV, cm/second	1587 ± 361	^†^ 1454 ± 202	1512 ± 237	1788 ± 483	0.01
CIMT, mm	0.67 ± 0.12	0.64 ± 0.14	0.67 ± 0.13	0.71 ± 0.09	0.201
Carotid plaque, *n* (%)	21 (40.4)	4 (23.5)	6 (33.3)	11 (64.7)	0.038

Data are presented as *n* (%) or mean ± standard deviation, as appropriate. OCTA, optical coherence tomography angiography; ASCVD, atherosclerotic cardiovascular disease; STEMI, ST-elevation myocardial infarction; BNP, brain natriuretic peptide; eGFR, estimated glomerular filtration rate; HDL-C, high density lipoprotein cholesterol; LDL-C, low density lipoprotein cholesterol; LVEF, left ventricular ejection fraction; baPWV, brachial-ankle pulse wave velocity; CIMT, Carotid intima–media thickness. ^†^ Post hoc *p*: low versus high group statistically significant *p* < 0.05. * Post hoc *p*: low versus intermediate group statistically significant *p* < 0.05. ^‡^ Post hoc *p*: intermediate versus high group statistically significant *p* < 0.05.

**Table 2:** Baseline characteristics in the three groups according to the average size of choriocapillaris measured by OCTA.

	Overall Patients (*n* = 60)	Low Average Size of CCP (*n* = 20)	Intermediate Average Size of CCP (*n* = 20)	High Average Size of CCP (*n* = 20)	*p*-Value
Clinical characteristics					
Age, years	59 ± 12	^†^ 53 ± 8	^‡^ 56 ± 12	68 ± 11	<0.001
Male sex, *n* (%)	54 (90)	18 (90)	18 (90)	18 (90)	1
Systolic blood pressure, mmHg	136 ± 31	137 ± 38	132 ± 27	140 ± 29	0.752
Diastolic blood pressure, mmHg	83 ± 19	86 ± 21	83 ± 19	82 ± 17	0.775
Heart rate, beats/min	78 ± 23	78 ± 20	77 ± 33	80 ± 14	0.935
Current smoker, *n* (%)	37 (61.7)	16 (80)	12 (60)	9 (45)	0.088
STEMI, *n* (%)	37 (61.7)	13 (65)	13 (65)	11 (55)	0.842
Killip class	1.5 ± 1.0	1.6 ± 1.2	1.4 ± 0.8	1.6 ± 1.1	0.821
Comorbidities, *n* (%)					
Hypertension, *n* (%)	27 (45)	1 (5)	11 (55)	15 (75)	<0.001
Diabetes mellitus, *n* (%)	17 (28.3)	5 (25)	4 (20)	8 (40)	0.45
Dyslipidemia, *n* (%)	14 (23.3)	0 (0)	7 (35)	7 (35)	0.006
Chronic kidney disease, *n* (%)	13 (21.7)	3 (15)	2 (10)	8 (40)	0.09
Ischemic stroke, *n* (%)	3 (5)	0 (0)	1 (5)	2 (10)	0.766
Laboratory findings					
Troponin I, ng/mL	3.2 ± 9.7	0.3 ± 0.5	6.2 ± 15.8	3.0 ± 5.0	0.163
BNP, pg/mL	260 ± 464	^†^ 94 ± 103	184 ± 206	501 ± 721	0.012
Creatinine, mg/dL	0.98 ± 0.42	0.92 ± 0.23	0.94 ± 0.35	1.07 ± 0.60	0.513
eGFR, mL/min/1.73 m^2^	87 ± 20	92 ± 17	90 ± 21	79 ± 21	0.103
Glucose level, mg/dL	168 ± 78	178 ± 102	155 ± 63	172 ± 64	0.617
Total cholesterol level, mg/dL	173 ± 43	^†^ 198 ± 46	167 ± 33	155 ± 38	0.003
Triglyceride level, mg/dL	155 ± 68	166 ± 63	160 ± 77	139 ± 64	0.409
HDL-C level, mg/dL	41 ± 9	40 ± 9	40 ± 8	42 ± 11	0.615
LDL-C level, mg/dL	97 ± 34	^†^ 117 ± 37	95 ± 21	81 ± 33	0.002
LVEF, %	51 ± 12	53 ± 10	50 ± 11	49 ± 15	0.548
baPWV, cm/second	1587 ± 361	1481 ± 204	1654 ± 417	1623 ± 413	0.328
CIMT, mm	0.67 ± 0.12	0.63 ± 0.10	0.69 ± 0.14	0.71 ± 0.11	0.162
Carotid plaque, *n* (%)	21 (40.4)	5 (26.3)	8 (42.1)	8 (57.1)	0.223
Cardiovascular risk score					
10-year ASCVD risk (%)	19.7 ± 17.0	^†^ 13.3 ± 8.3	16.5 ± 17.2	29.1 ± 19.5	0.007
GRACE score	107 ± 33	^†^ 99 ± 38	100 ± 27	123 ± 29	0.032
TIMI risk score	2.0 ± 1.2	^†^ 1.4 ± 0.7	1.9 ± 1.0	2.6 ± 1.5	0.009
REACH score CV death	12.4 ± 3.5	^†^ 10.3 ± 2.2	^‡^ 11.9 ± 3.0	15.0 ± 3.4	<0.001
REACH score CV event	14.4 ± 3.8	^†^ 12.6 ± 2.7	^‡^ 13.7 ± 3.5	17.0 ± 3.7	<0.001
SYNTAX score	15.8 ± 8.0	15.4 ± 8.1	15.4 ± 8.5	16.7 ± 7.6	0.834

Data are presented as *n* (%) or mean ± standard deviation, as appropriate. OCTA, optical coherence tomography angiography; CCP, choriocapillaris; STEMI, ST-elevation myocardial infarction; BNP, brain natriuretic peptide; eGFR, estimated glomerular filtration rate; HDL-C, high density lipoprotein cholesterol; LDL-C, low density lipoprotein cholesterol; LVEF, left ventricular ejection fraction; baPWV, brachial-ankle pulse wave velocity; CIMT, Carotid intima–media thickness; ASCVD, atherosclerotic cardiovascular disease; GRACE score, Global Registry of Acute Cardiac Events score; REACH score, Reduction of Atherothrombosis for Continued Health score; CV, cardiovascular; SYNTAX score, the residual Synergy Between Percutaneous Coronary Intervention With Taxus and Cardiac Surgery score. ^†^ Post hoc *p*: low versus high group statistically significant *p* < 0.05. ^‡^ Post hoc *p*: intermediate versus high group statistically significant *p* < 0.05.

**Table 3:** Univariate and multivariate logistic regression analysis to evaluate the association between the high average size of choriocapillaris flow void and baseline characteristics with 10-year ASCVD risk.

* Variables	Univariate		^‡^ Multivariate	
	OR (95% CI)	*p*-Value	Adjusted OR (95% CI)	*p*-Value
Chronic kidney disease, *n* (%)	4.667 (1.278–17.047)	0.020	–	–
BNP, pg/mL	1.003 (1.0005–1.006)	0.020	1.003 (1.0001–1.005)	0.044
eGFR, mL/min/1.73 m^2^	0.972 (0.945–0.999)	0.046	–	–
^†^ 10-year ASCVD risk	1.053 (1.015–1.092)	0.006	1.044 (1.006–1.084)	0.024

OR, odds ratio; ASCVD, atherosclerotic cardiovascular disease; BNP, brain natriuretic peptide; eGFR, estimated glomerular filtration rate. ^†^ 10-year ASCVD risk includes age, sex, race, systolic blood pressure, diastolic blood pressure, total cholesterol level, high-density lipoprotein cholesterol level, low-density lipoprotein cholesterol level, medical history of diabetes mellitus, treatment of hypertension, statin medication, and aspirin. * Included variables were 10-year ASCVD risk and baseline characteristics with statistical significance, except for variables reflected in 10-year ASCVD risk scores. ^‡^ Backward elimination with a threshold of *p* = 0.1 was used as the criterion for retention of a variable in the model.

**Table 4:** Univariate and multivariate logistic regression analysis to evaluate the association between the high average size of choriocapillaris flow void and baseline characteristics with GRACE score.

* Variables	Univariate		^‡^ Multivariate	
	OR (95% CI)	*p*-Value	Adjusted OR (95% CI)	*p*-Value
Hypertension, *n* (%)	7.000 (2.072–23.645)	0.002	6.763 (1.741–26.274)	0.006
Chronic kidney disease, *n* (%)	4.667 (1.278–17.047)	0.020	4.503 (0.915–22.160)	0.064
BNP, pg/mL	1.003 (1.0005–1.006)	0.020	–	–
Total cholesterol level, mg/dL	0.981 (0.965–0.997)	0.022	–	–
LDL-C level, mg/dL	0.973 (0.954–0.994)	0.011	–	–
^†^ GRACE score	1.024 (1.005–1.044)	0.013	1.021 (1.001–1.042)	0.042

OR, odds ratio; GRACE score, Global Registry of Acute Cardiac Events score; CI, confidence interval; BNP, brain natriuretic peptide; LDL-C, low-density lipoprotein cholesterol. ^†^ GRACE score includes age, heart rate, systolic blood pressure, Killip class (or use of diuretics), serum creatinine level (or presence of acute kidney injury), elevation of cardiac biomarkers, ST-segment deviation on electrocardiography, and cardiac arrest at admission. * Included variables were GRACE score and baseline characteristics with statistical significance, except for variables reflected in GRACE score. ^‡^ Backward elimination with a threshold of *p* = 0.1 was used as the criterion for retention of a variable in the model.

**Table 5:** Univariate and multivariate logistic regression analysis to evaluate association between high average size of choriocapillaris flow void and baseline characteristics with REACH score.

* Variables	Univariate		^‡^ Multivariate	
	OR (95% CI)	*p*-Value	Adjusted OR (95% CI)	*p*-Value
Hypertension, *n* (%)	7.000 (2.072–23.645)	0.002	–	–
Chronic kidney disease, *n* (%)	4.667 (1.278–17.047)	0.020	–	–
BNP, pg/mL	1.003 (1.0005–1.006)	0.020	1.002 (0.999–1.005)	0.147
Total cholesterol level, mg/dL	0.981 (0.965–0.997)	0.022	–	–
LDL-C level, mg/dL	0.973 (0.954–0.994)	0.011	–	–
eGFR, mL/min/1.73 m^2^	0.972 (0.945–0.999)	0.046	–	–
^†^ REACH score CV death	1.499 (1.198–1.876)	<0.001	1.413 (1.121–1.782)	0.003

OR, odds ratio; REACH score, REACH score, reduction of atherothrombosis for continued health score; CI, confidence interval; BNP, brain natriuretic peptide; LDL-C, low-density lipoprotein cholesterol; eGFR, estimated glomerular filtration rate; CV, cardiovascular. ^†^ REACH score includes age, sex, race, body mass index, smoking status, medical history of diabetes mellitus, number of diseased vascular beds, cardiovascular events in the past year, medical history of congestive heart failure, presence of atrial fibrillation, use of statins, and use of aspirin. * Included variables were REACH score and baseline characteristics with statistical significance, except for variables reflected in REACH score. ^‡^ Backward elimination with a threshold of *p* = 0.1 was used as the criterion for retention of a variable in the model.

## Data Availability

The data that support the findings of this study are available from the corresponding author upon reasonable request.
